# Scaling up the delivery of refractive error services within a district health system: the KwaZulu-Natal, South Africa experience

**DOI:** 10.1186/1472-6963-13-361

**Published:** 2013-09-27

**Authors:** Kovin S Naidoo, Kesi Naidoo, Yashika Maharaj, Prasidh Ramson, Diane Wallace, Reshma Dabideen

**Affiliations:** 1 Brien Holden Vision Institute, Durban, South Africa; 2 African Vision Research Institute (AVRI), UKZN, Durban, South Africa; 3 University of KwaZulu-Natal, Durban, South Africa; 4 ORBIS International, Cape Town, South Africa

**Keywords:** Public health, Primary eye health, Refractive error, Service delivery, District health system

## Abstract

**Background:**

In South Africa, the health service is based on a Primary Health Care (PHC) philosophy with the District Health System (DHS) as the locus of delivery. However eye care services, particularly primary eye care, refractive error and low vision, have not been prioritised accordingly. Hence the aim of the Giving Sight to KwaZulu-Natal (GSKZN) project was to integrate the delivery of eye care services into the district health system, with emphasis on addressing the need for uncorrected refractive error and low vision services.

The project was implemented in the KwaZulu-Natal province, South Africa, to scale up the delivery of refractive error services utilising a four pronged approach; including advocacy, human resource development, equipment provision and research.

**Methods:**

This paper is a description of the project and a retrospective analysis of data received through the course of the project from July 2007 to June 2011. Data were collected from training registers, equipment schedules and service delivery reports from institutions. Reports from the data base were then analysed and achievements in training and trends in service delivery were determined.

**Results:**

Over a four year period (July 2007 and July 2011) 1004 persons received training in rendering eye health services appropriate to their level of deployment within the DHS. During the course of the project, these 1004 persons examined 1 064 087 patients. Furthermore, the total number of clinics offering primary eye care, refractive error and low vision services increased from 96 (10%) to 748 (76%). With increased numbers of PHC Nurses trained in primary eye care, a subsequent decrease of 51.08 percent was also observed in the number of patients seeking services at higher levels of care, thus streamlining eye health service delivery.

**Conclusion:**

This project has shown that scaling up can occur in delivering eye health services within a health district, through a multi-faceted approach that encompasses focused training, advocacy, development of appropriate infrastructure and the development of referral criteria with clear guidelines for the management of patients.

## Background

### Refractive error

Six hundred and forty (640) million people worldwide are estimated to be either blind or visually impaired due to uncorrected refractive error [1,2]. Refractive error refers to the measure of a person’s short-sightedness (myopia), far-sightedness (hyperopia) and/or astigmatism (corneal curvature). In the absence of any organic cause for poor vision, refractive error can easily be corrected with a pair of spectacles [3]. Uncorrected refractive error is the second leading cause of blindness after cataract and the main cause of low vision. Overall, it is the cause of almost half of all visual impairment [3]. Annually, the global economy loses $269 billion in productivity due to uncorrected refractive error [4].

Visual disability resulting from uncorrected refractive error has a major impact on the life of the affected person as they are more likely to be excluded from education opportunities, suffer from isolation and have fewer employment opportunities thus perpetuating the vicious cycle of poverty [5]. A Refractive Error and Visual Impairment study (RESCA) conducted in Durban, South Africa in 2003, found that of those children with reduced vision, 63% was due to refractive error. In addition, the study found that only 20% of the children that needed spectacles had them, indicating that four out of every five children with vision problems were unnecessarily visually impaired or blind [6].

### The Giving Sight to KwaZulu-Natal project

The District Health System (DHS), which has been identified as an appropriate building block for a National Health System by the World Health Organisation (WHO), is a framework for the delivery of Primary Health Care (PHC) - a model promoted as a solution to creating access at a local level [7]. In South Africa, the DHS has been the cornerstone of government policy on health; however the delivery of eye health services within the DHS has been poorly defined. There is a lack of comprehensive programmes to ensure that the various cadres within eye health are integrated into a seamless unit of service delivery and appropriate referral. Thus the incorporation of eye health into the DHS was an urgent priority in ensuring appropriate delivery of eye health services, more specifically, refractive error and low vision services.

South Africa has a population of 50.59 million (South African census 2011) [8]. The project was implemented in the province of KwaZulu-Natal (KZN) with a population of 10 819 130. Forty-three percent (43%) of the KZN population lives in urban areas. The province consists of eleven health planning regions [9].

Currently in South Africa, the role of various eye health personnel within DHS has not been clearly defined [10]. Whilst the policy for the DHS demands that integrated and essential PHC services are available to the entire population at the first point of contact, these basic service packages did not include eye health services. Therefore, the Brien Holden Vision Institute (formerly the International Center for Eye Care Education) supported by Standard Chartered Bank’s “Seeing is Believing Campaign”, embarked on a project to integrate the delivery of eye health services, with emphasis on uncorrected refractive error and low vision, into the DHS. This development was designed to scale up the delivery of refractive error and low vision services within the DHS of South Africa.

### Design and implementation of the giving sight project delivery system

In South Africa, eye health services are delivered from PHC Clinics with referral pathways going up to Quaternary level hospitals if necessary [10]. Eye health workers active in the province include Ophthalmic Nurses, Optometrists and Ophthalmologists. To scale up the delivery of refractive error services, the following method was utilised:

1. Advocacy for the delivery of primary eye care, refractive error and low vision services within all levels of the DHS.

2. Training and deployment of the appropriate human resources.

3. Development of infrastructure to enable the delivery of services.

4. Development of a data collation and management system for the program.

1. *Advocacy*

The KwaZulu-Natal Department of Health (KZN DoH) was identified as the key stakeholder of the project. Stakeholder engagement on the value of developing a sustainable strategy for the delivery of refractive error services within the DHS was conducted. Consultative meetings included relevant persons within the KZN DoH. This resulted in the appointment of the steering committee responsible for the design of the project and development of the Memorandum of Understanding (MOU). The Health Operation Committee was further engaged to inform senior provincial and district managers about the project, inviting their input on proposed project plans. All district eye care coordinators were then engaged to discuss implementation of the project; these project implementation plans were subsequently reviewed at district meetings to address district specific needs.

2. *Development of Appropriate Human Resources*

A two-fold approach was utilised in the training component of the project. Firstly, eye care cadres who were already delivering refractive error services (ophthalmic nurses and optometrists) were appropriately up skilled. Secondly, primary health care nurses, who did not have previous primary eye care training were trained (Table 1). The training program for the PHC nurses was based on the development of competencies as defined in the National Guideline on Management and Control of eye conditions at a primary level [11].

To enable the PHC nurses to act as effective “gate keepers” of the eye care pathway, core competencies such as visual acuity measurements and the detection of basic anterior segment pathological conditions were developed. KZN DoH provided a total of 924 PHC nurses selected from Health Districts in need of eye care services, to be trained in primary eye care. Of those trained, 75 PHC nurses received additional training enabling them to conduct posterior segment exams using a direct ophthalmoscope, usually part of the skills set of ophthalmic nurses. These nurses were selected from Health Districts where there were no ophthalmic nurses (ON), who would otherwise screen for posterior segment disease. This was seen as an interim measure to address the lack of ophthalmic nurses in such areas.

3. *Development of appropriate infrastructure*

Recommendations were made to the KZN DoH Infrastructure Development Unit to allocate appropriate space for eye clinics in the future planning of institutions. In addition, equipment, as shown in Table 2 was provided to all cadres trained to ensure that they were able to adequately function at the recommended level within the DHS. Equipment donated to the various health institutions was taken into the institution’s stock management system to ensure that future maintenance and replacement would be the responsibility of the health institution.

An assistive device delivery system was developed to facilitate affordable access for patients. An optical workshop was established at a central location and the supporting logistics to deliver assistive devices to patients was developed. Ready-made readers, single vision and bifocal spectacles with a multi-tiered pricing option were available to patients. Low vision devices were also available. All school children and indigent adults with significant vision impairment were provided with their assistive devices at no cost, subsidised by the sale to others.

4. *Data acquisition and management system of the program.*

Once trained, all trainees were requested to report data on a monthly basis for the purpose of monitoring the program. They were requested to collate and report on the following:

1. Number of patients examined

1. Number of patients who received treatment

1. Number of patients referred for further treatment

The KZN DoH had not collected eye health data at primary health care level prior to the implementation of the project and the data collection tools at secondary levels of health care had not been standardised. Therefore data collection forms were specifically designed to enable primary health care nurses, ophthalmic nurses and optometrists to record statistics of patients examined, treated and referred on a monthly basis. The standardised tool was hand recorded and submitted to the project office and DHIS office.

**Table 1:** Details of trainings conducted

**Cadre trained**	**Key competencies developed**	**Duration of training**
Primary Health Care Nurses *Part 1*	• Measurement of Visual acuity	5 days
	Identification of basic eye pathological conditions	
Primary Health Care Nurses *Part 2 (Advanced Training offered to a percentage of those that completed Part 1)*	• Ophthalmoscopy	5 days
	Identification of basic posterior segment diseases	
Ophthalmic Nurses	• Basic Refraction	5 days
	Diagnosis & Management of Ocular Disease	
Optometrists	• Low Vision Assessments	5 days
	• Treatment of Binocular Vision Anomalies	2 days
	• Posterior Segment Pathology	

**Table 2:** Equipment supplied per cadre trained

	**Pen - light torch**	**Pinhole & occluder**	**VA chart**	**Ophthalmoscope**	**Retinoscope**	**Trial case & frame**	**Slit lamp**	**20 D & 90 D lens**	**Low vision starter sets**
**Primary Eye Care Nurses**	X	X	X						
**Ophthalmic Nurses**	X	X	X	X	X	X			
**Optometrists**	X	X	X	X	X	X	X	X	X

## Methods

This paper is a retrospective analysis of data received through the course of the project from July 2007 to June 2011. An electronic capturing and reporting database application using Microsoft Visual Studio and Microsoft SQL Server was developed. All data received from training registers, equipment schedules, service delivery reports and assistive devices supplied was entered onto the data base. Reports from the data base were then exported to Excel 2007 for analysis.

The data was collected during the program as follows:

1. Records of attendance at training sessions

All attendees at training sessions completed a daily attendance register providing their name, designation, institution and service area. This included professional nurses, ophthalmic nurses and optometrists. Attendance registers were then captured onto Excel 2007 spread sheets disaggregated by cadre trained and training session.

2. Service delivery

Monthly records of the patients examined at each clinic were reported either through direct submission to the project office or through the DHIS. The project office received quarterly reports from the DHIS. The indicators reported for professional nurses, including ophthalmic nurses were:

a. Number of patients examined

a. Number of patients treated

a. Number of patients referred

The DoH did not provide individual patient records to the project team as these were confidential and stored at the health institution visited; only aggregated reports were provided. Monthly reports of the number of assistive devices supplied were collected.

## Results

Between July 2007 and July 2011, 1004 persons were trained in the delivery of refractive error services appropriate to the level at which they functioned within the DHS as shown in Table 3.

**Table 3:** Number of persons trained per level

**Level**	**Cadre**	**2007/2008**	**2008/2009**	**2009/2010**	**Total trained**
PHC	Primary Health Care Nurses - Part 1	92	540	292	924
	Primary Health Care Nurses - Part 2			75	75
CHC; District; Regional and Tertiary	Ophthalmic Nurses			42	42
	Optometrists		38		38
				*Total Trained*	1004

Collectively, these 1004 trained persons examined a total of 1 064 087 patients over the project period. The breakdown of the outputs per cadre is shown in Table 4.

**Table 4:** Numbers of patients examined by cadres per level

**Level**	**Cadre**	**Jul 07 - Dec 07**	**Jan 08 - Jun 08**	**Jul 08 - Dec 08**	**Jan 09 - Jun 09**	**Jul 09 - Dec 09**	**Jan 10 -June 10**	**Jul 10 - Dec 10**	**Jan 11 - to Jun 11**	**TOTALS**
PHC	PHC Nurses	4417	8736	20373	51820	63291	84346	71153	99422	403558
District	Ophthalmic Nurses	40867	51087	49740	44576	39360	43419	26534	36444	332027
	Optometrists	4393	6527	6570	15774	18466	23648	14828	15973	106179
Regional	Ophthalmic Nurses	9006	27272	31856	31985	9387	4756	6092	4825	125179
	Optometrists	2806	1689	4617	5961	7011	9821	6001	8241	46147
Tertiary	Ophthalmic Nurses	6061	5865	3026	326	5202	5124	5474	5797	36875
	Optometrists	1715	2136	667	1616	1531	2051	1727	2320	13763
Quaternary	Optometrists	0	7	0	0	150	189	0	13	359
TOTALS		69265	103319	116849	152058	144398	173354	131809	173035	1064087

**Figure 1:**
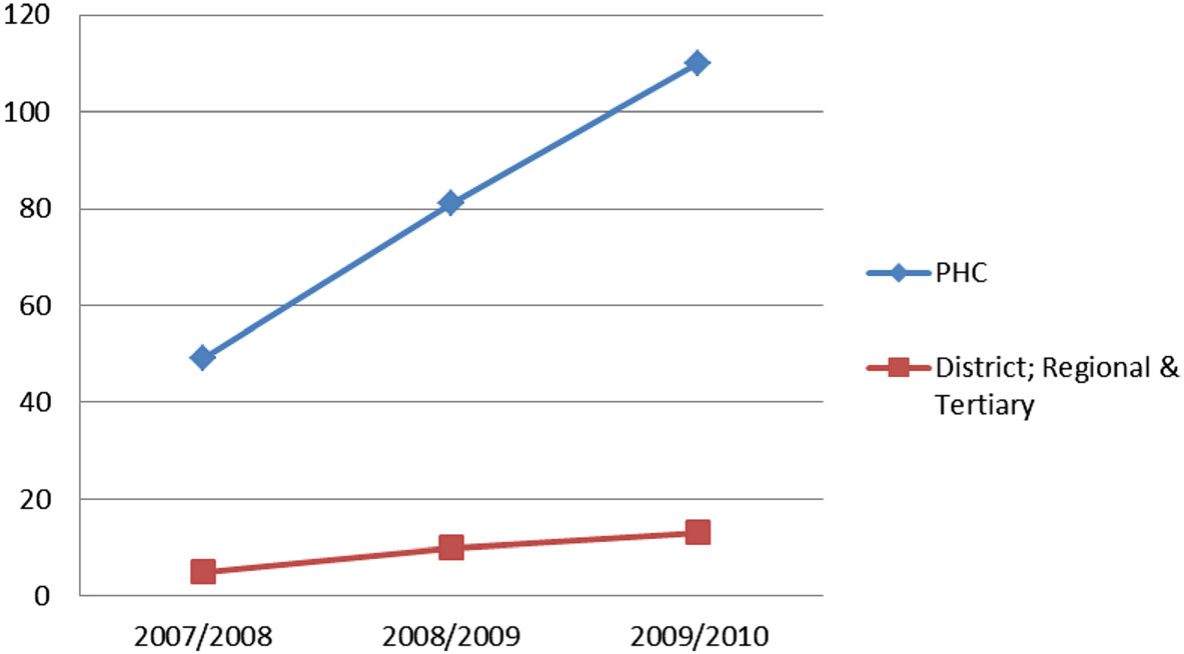
Growth in the number of clinics that offer refractive error services over the project period.

As the project progressed, there was a growth in the number of clinics within the KZN DoH that was offering refractive error services (Figure 1). There are 982 clinics in KwaZulu-Natal (inclusive of mobile and fixed visiting points), and out of those only 96 were reporting eye health data, and after the program that figure increased to 748. PHC clinics providing PEC increased from 42 to 613 and other clinics (district, regional and tertiary) increased from 54 to 135. The coverage increased from 10% to 76%.

**Figure 2:**
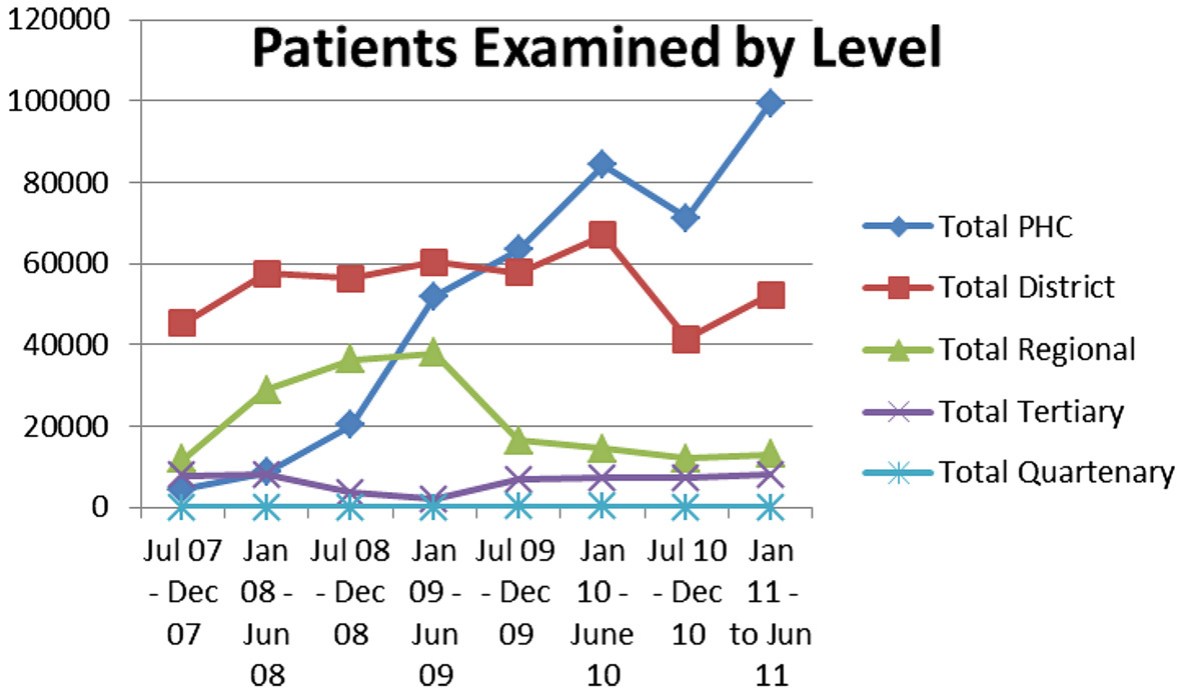
Numbers of patients examined per level of the DHS over the project period.

**Figure 3:**
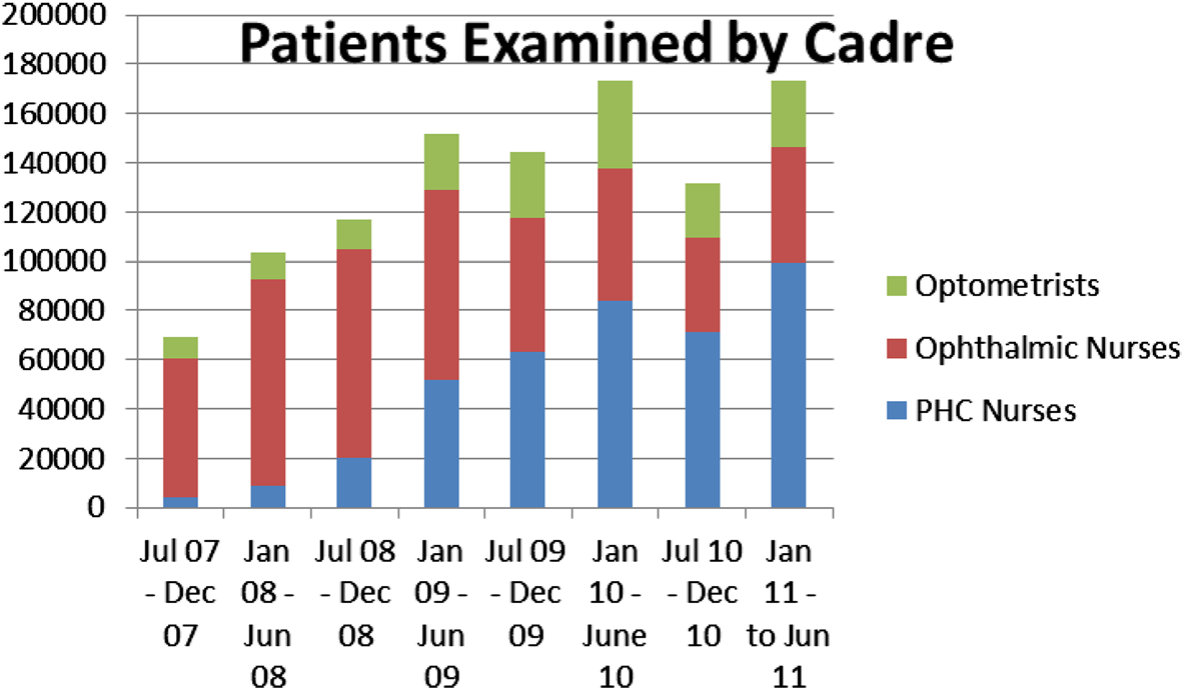
The number of patients examined per cadre during the project period.

With an increase in the number of PHC Nurses being trained in primary eye care, a corresponding increase in the number of patients being examined at a primary health care (PHC) level was observed (Figure 2), indicating that nurses trained were conducting primary eye care at their respective institutions and implementing skills learned during the training. There was a corresponding decrease in the number of patients seen at district, regional and tertiary levels as shown in Figure 3.

The number of patients examined by PHC nurses showed an increase from approximately 5000 in the first period to almost 100 000 in the last reporting period. Optometry services also increased during the project period reflecting more than twice the number of patients accessing this service. The total number of patients examined also increased from just over 60 000 to over 160 000 representing an increase of approximately two and half times.

Before commencement of the project, there were 6 optometrists serving 41 health institutions within the KZN DoH. Through this project, the number of optometrists increased to 38 optometrists serving 117 institutions– some funded directly by the project, and others appointed by KZN DoH as a result of the project’s advocacy efforts. Currently there are 38 optometrists in the public sector with government employing 32. Skills audits were performed to identify additional areas of training required. An overwhelming majority of optometrists indicated the need for assistance in the areas of Low Vision, Binocular Vision and Posterior Segment pathology.

## Discussion

Prior to the implementation of the project, refractive error services were offered at only a few public health institutions in KwaZulu-Natal – primarily due to the fact that there were only 6 full time optometrists working within the KZN DoH at the inception of the project. Furthermore the majority of PHC nurses were not delivering primary eye care, but merely referring all “eye cases” along the referral pathway, needlessly creating a backlog at the District and Regional Level.

Training of ONs in the province was discontinued by the KZN DoH in 2002 [Zimu N. 2002, personal communication]. To compound the situation, many ONs were deployed to other areas of clinical care as well as into clinic and hospital management roles. At the time of the project inception there were only 46 active ONs working in KZN. As this cadre had already received training in the delivery of refractive error services (as part of their ON training), a skills audit was conducted to identify areas in which the nurses felt they required further training. The results indicated a need for further training in basic refraction and the diagnosis of posterior segment diseases. As such, a post evaluation needs to be conducted at a later stage to determine the impact of the project’s training on the quality of eye care delivered by ONs.

Despite the development of tools and systems for data collection from all cadres trained, data collection remained a challenge. Extensive telephonic contact with trainees yielded varying levels of success in acquiring data. Optometrists and ONs reports were received fairly regularly; however reporting from PHC clinics was limited and very sporadic. As such, attempts were made to add eye care indicators into the DHIS, an existing data Management System utilized by the KZN DoH. This initiative proved successful.

The need for continuous advocacy for eye care services within the KZN DoH beyond the project lifecycle is critical to the maintenance and continued development of refractive error services established through this project. To this end, provincial forums for ONs and Optometrists have been established. An enabling factor in this regard was the existence of a policy for the formation of forums in the KZN DoH. These forums have been successfully launched, and the KZN optometry forum has also affiliated itself with the National Public Sector Optometry Forum in South Africa.

The role of community based cadres, particularly community Health workers and Traditional healers was not developed extensively in the project. The Community Health Worker (CHW) program is not uniformly developed in the province and this cadre was therefore not included in the capacity development initiative. However, a role in health promotion and case finding has been identified for these cadres and presents an opportunity for further development of eye care and refractive error services.

The increase in the percentage of patients seen at PHC level and the corresponding decrease in percentage of patients seen at District and Regional Level is of importance and is likely due to the presence of the newly trained PHC nurse within the eye care referral system. Previously patients would have needed to travel to the district and regional institutions to access the closest eye care service which was provided by ophthalmic nurses or an optometrist. These patients often needed treatment for simple bacterial or allergic conjunctivitis, which can now be managed by the PHC nurse at their local clinic resulting in a reduction in the percentage of patients reporting at secondary and tertiary level respectively. This allows for the more efficient use of the skills of personnel at different levels of the DHS.

A limitation of the analysis is a lack of information to determine the quality of service being provided by the different cadres. We were also unable to track the numbers that did not present after referrals and the non-compliance associated with this. Some patients were counted both at the primary and secondary referral sites as we were not able to determine from the data who were referred and who were not.

It will also be useful to determine the patients’ perception of the service. Furthermore an assessment of the quality of training rendered needs to be conducted.

## Conclusion

This project has indicated that scale up of refractive error services can occur in a health district through a multi-faceted approach that encompasses wide spectrum training, advocacy, appropriate infrastructure and referrals with clear guidelines for the management of patients. It has shown that within an African context the District Health System offers an alternative to models of delivery implemented in other parts of the world that have been successfully working outside the public health system. It must however be noted that while up scaling is possible, it is dependent on the effective collaboration of the respective governments. With the number of patients served within this particular project, we can assume that the development of refractive error services significantly increased the number of patients attending public sector eye clinics. Therefore the district health system can be an appropriate vehicle for the up scaling of services.

## Abbreviations

DHS: District Health System; PHC: Primary Health Care; WHO: World Health Organisation; RESCA: Rapid Assessment of Refractive Error in School Children; KZN: KwaZulu-Natal; KZN DoH: KwaZulu-Natal Department of Health; The Institute: Brien Holden Vision Institute; PEC: Primary Eye Care; DHIS: District Health Information System; ON: Ophthalmic Nurse; CHW: Community Health Workers.

## Competing interests

The authors declare that they have no competing interests.

## Authors’ contributions

KSN: Conception; design & intellectual contribution; manuscript drafting. KN: Study design; intellectual contribution. YI: Intellectual contribution; manuscript drafting. PR: Analysis of Data; intellectual contribution. DW: Intellectual contribution. RD: Intellectual contribution. All authors read and approved the final manuscript.

## Pre-publication history

The pre-publication history for this paper can be accessed here:

http://www.biomedcentral.com/1472-6963/13/361/prepub
